# Enantioselective copper catalysed, direct functionalisation of allenes *via* allyl copper intermediates

**DOI:** 10.1039/c7sc01968h

**Published:** 2017-06-08

**Authors:** Alexander P. Pulis, Kay Yeung, David J. Procter

**Affiliations:** a School of Chemistry , University of Manchester , Oxford Rd , Manchester , M13 9PL , UK . Email: alexander.pulis@manchester.ac.uk ; Email: david.j.procter@manchester.ac.uk

## Abstract

The direct functionalisation of allenes under copper catalysis enables efficient access to enantioenriched, densely functionalised molecules. In this review we explore the breadth and depth of a versatile reaction manifold, which involves the element-cupration of allenes to generate allyl copper intermediates that are subsequently coupled with diverse arrays of electrophiles.

## Abstract 2

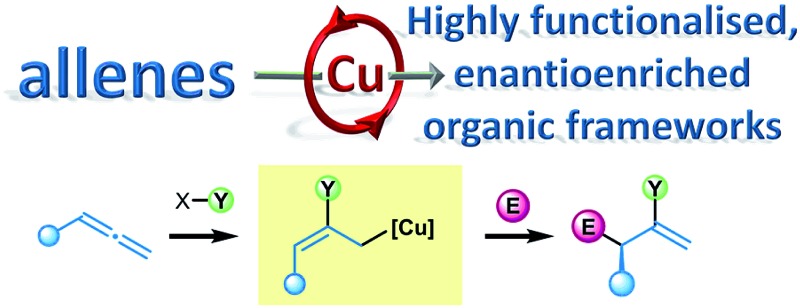
We explore the breadth of the copper catalysed functionalisation of allenes, which enables efficient access to enantioenriched, densely functionalised molecules.

## 1 Introduction

The formation of densely functionalised organic molecules from readily available feedstocks is a critical activity in synthetic science. Such processes are particularly powerful when they operate under mild conditions, utilise readily available inexpensive nontoxic catalysts, and selectively deliver single enantiomers.

Allenes, once only an academic curiosity due to their unique geometry, have become versatile building blocks in the synthesis of complex molecules;^1^ more reactive than alkenes and alkynes, they allow mild and atom efficient transformations, and are ideal substrates for asymmetric catalysis. Possessing 1,2-orthogonal double bonds, the use of allenes is not without challenges since regio- and chemoselective issues can arise and 1,3-disubstituted allenes are chiral entities. Despite this, they have become popular starting materials in multicomponent synthesis since metal-functionalisation of one carbon–carbon double bond often leads to an allyl metal species that can be subsequently reacted further with a variety of coupling partners.^2^

**Scheme 1:**
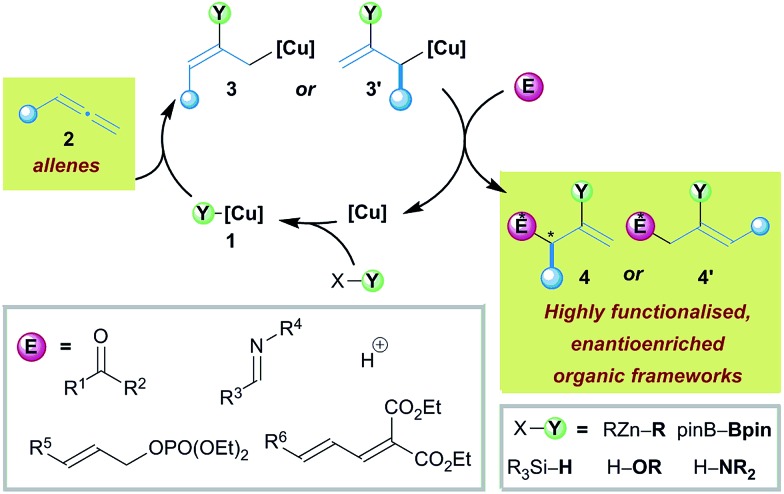
Copper catalysed functionalisation of allenes allows access to diverse collections of enantioenriched organic frameworks.

In recent times, copper catalysis has offered an inexpensive, environmentally friendly alternative to the use of precious metals.^3,4^ Surprisingly, only recently have allenes and copper catalysts, and the resultant *in situ* generated versatile allyl copper intermediates, been allied to address the challenges of modern enantioselective synthesis (Scheme 1).^5^ The general mechanism for the copper catalysed functionalisation of allenes proceeds *via* initial formation of a copper–element complex **1**, for example a copper–boryl complex. Intermediate **1** then allows the direct functionalisation of an allene **2** *via* element-cupration,^3a,6^ which generally occurs at the least hindered site of the allene to generate an allyl copper^7^ species **3**. Metalotropic rearrangement of **3** can, in some cases, result in the formation of isomerised allyl copper **3′**. The resultant functionalised allyl coppers **3** and **3′** subsequently couple with various electrophiles through either the α- or γ-positions to selectively form densely functionalised, enantioenriched products **4** or **4′**. Although the regioselectivity in the initial element cupration and subsequent addition to electrophiles adds a further layer of complexity to the challenges of generating highly functionalised molecules in a stereodefined manner from allene feedstocks, remarkably high selectivity has been described. In this sequence, stereochemistry in the products may arise from the original allene carbon skeleton or from a prochiral electrophile, or in some cases from both.

Allyl coppers (*cf.* **3**) and the analogous propargyl and allenyl coppers utilised in similar enantioselective processes, can also be formed *via* cupration of related unsaturated carbon frameworks, such as in the works of Hoveyda,^8^ Buchwald,^9^ and Shimizu and Kanai^10^ involving enynes, and Cao and Liao^11^ employing 1,3-dienes, in addition to processes involving transmetalation.^12–15^

Allenes have proven versatile starting materials for the *in situ* generation of allyl copper intermediates (*cf.* **3**) by virtue of the breadth of multifunctionalisation reactions reported, and great effort has been invested by research groups worldwide in exploring the potential of this reaction manifold. In this review, we will analyse developments in the enantioselective, copper-catalysed direct functionalisation of allenes that involve allyl copper intermediates, and enable the efficient construction of highly versatile molecular architectures.

## 2 Copper catalysed enantioselective functionalisation of allenes

In this section we will detail the diverse array of enantioselective multifunctionalisation reactions of allenes under copper catalysis that proceed through allyl copper intermediates. For each reaction, we will discuss mechanistic features that underpin the key processes, include selected examples from the scope, and where possible, highlight the versatility of the products that are generated.

**Scheme 2:**
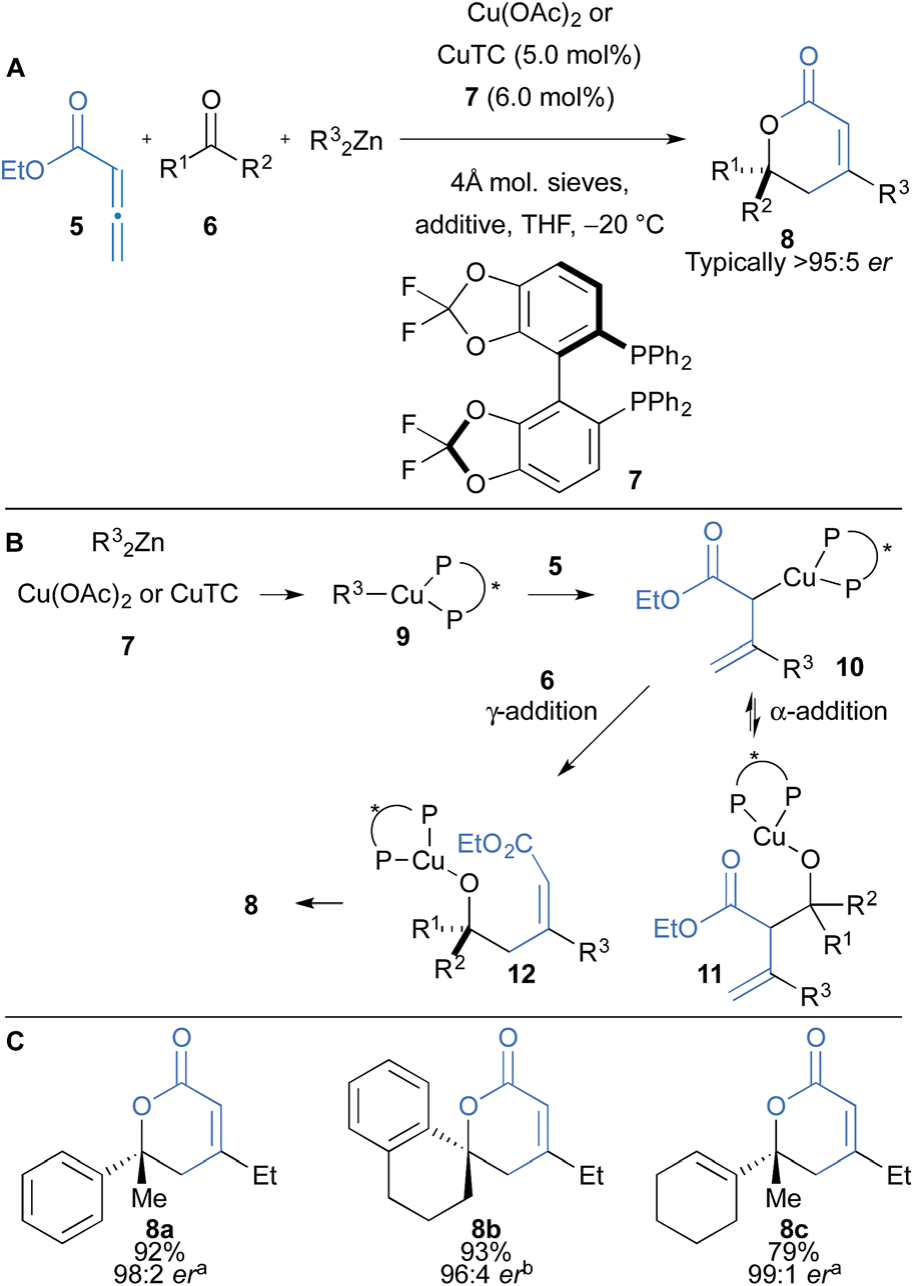
Kanai and Shibasaki's carbocupration of allenic esters and subsequent coupling to ketones. CuTC = copper thiophene-2-carboxylate; additive = DMSO^a^, HMPA^b^, or Ph_2_SO.

Kanai and Shibasaki reported the copper catalysed functionalisation of allenic esters **5** in a multicomponent coupling with ketones **6** and dialkyl zincs that delivers δ-lactones **8** with excellent enantioselectivity (Scheme 2).^16^ The reaction proceeds *via* formation of a chiral-phosphine alkyl copper(i) complex **9** which allows for carbocupration of allenic ester **5** forming allyl copper **10** (Scheme 2B). The reaction of **10** with ketones follows two courses: kinetic α-addition to give aldolate **11**, or γ-addition to give **12**. Indeed, mixtures of both **11** and **12** are initially formed, but retroaldol in **11** renders the α-addition pathway reversible, and the process selectively delivers the desired lactone product **8**. The addition of coordinating additives, such as sulfoxides or HMPA, facilitated the retro-aldol, and was crucial for high yields. The reaction scope was broad and, interestingly, even tolerated the use of α,β-unsaturated ketones (Scheme 2C).

**Scheme 3:**
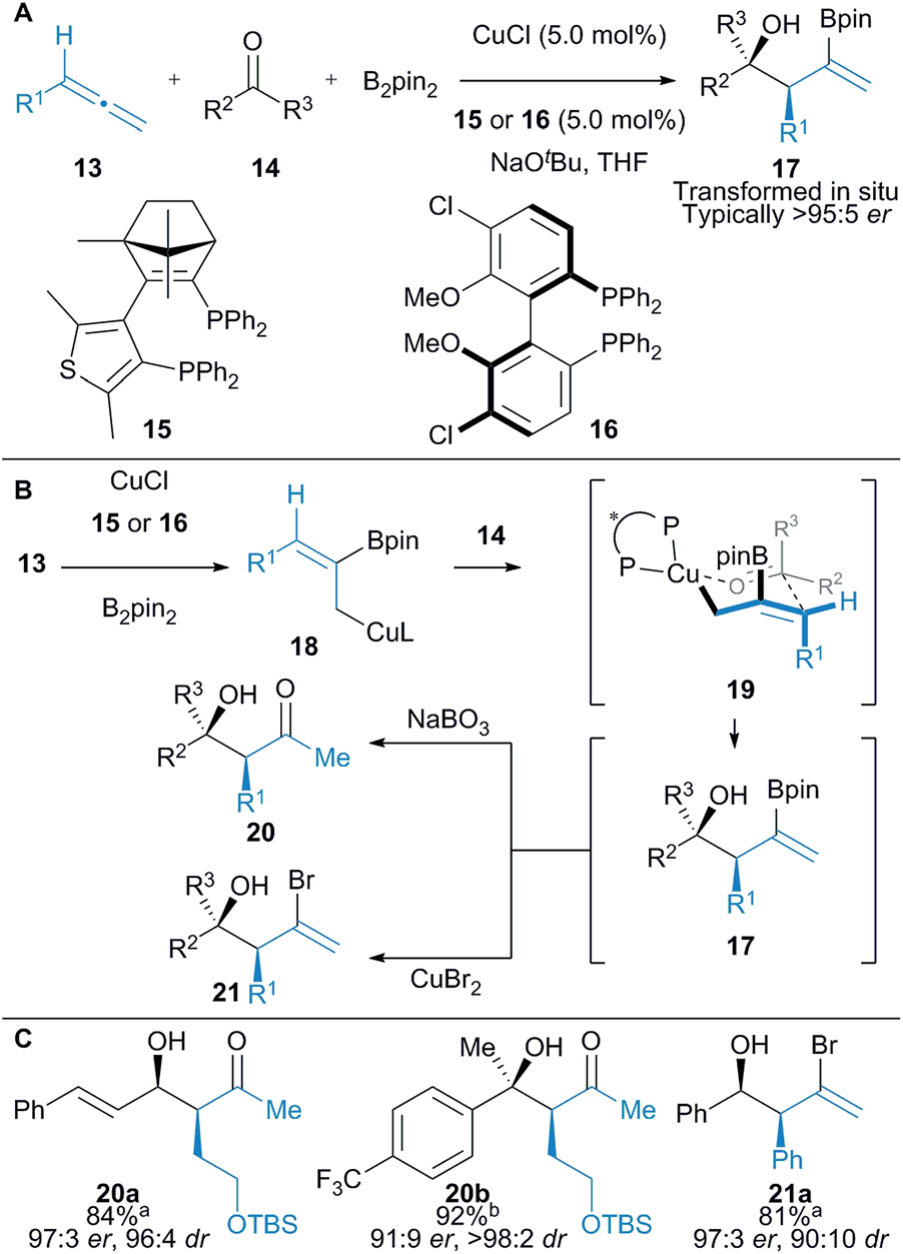
Hoveyda's borocupration of allenes and subsequent coupling to aldehydes and ketones. Enantiomeric ratio given for the major diastereomer. ^a^Ligand **15** was used at 4 °C; ^b^ligand **16** was used at 22 °C.

Hoveyda and co-workers described the enantioselective union of aldehydes or ketones (**14**) with aryl or alkyl substituted allenes (**13**) and B_2_pin_2_ (Scheme 3).^17^ Borocupration of the allene **13** generates *in situ* allylcopper **18**, which is trapped with an aldehyde or ketone to afford highly functionalised vinyl boronates **17**. Syn products are obtained *via* a proposed 6-membered transition state structure **19**, where the substituent on the aldehyde is in a pseudoequatorial position (Scheme 3B). Vinyl boronates **17** were not isolated and were oxidised or brominated *in situ* to give isolable β-hydroxyketones **20** or alkenyl bromides **21**, respectively. Enantioselective addition to ketones allowed access to tertiary alcohols (*cf.* **20b**) and interestingly, when α,β-unsaturated ketones were employed (*cf.* **20a**), 1,2-allylation was almost exclusively observed despite potential competing boryl–copper and allyl copper **18** conjugate addition pathways.

**Scheme 4:**
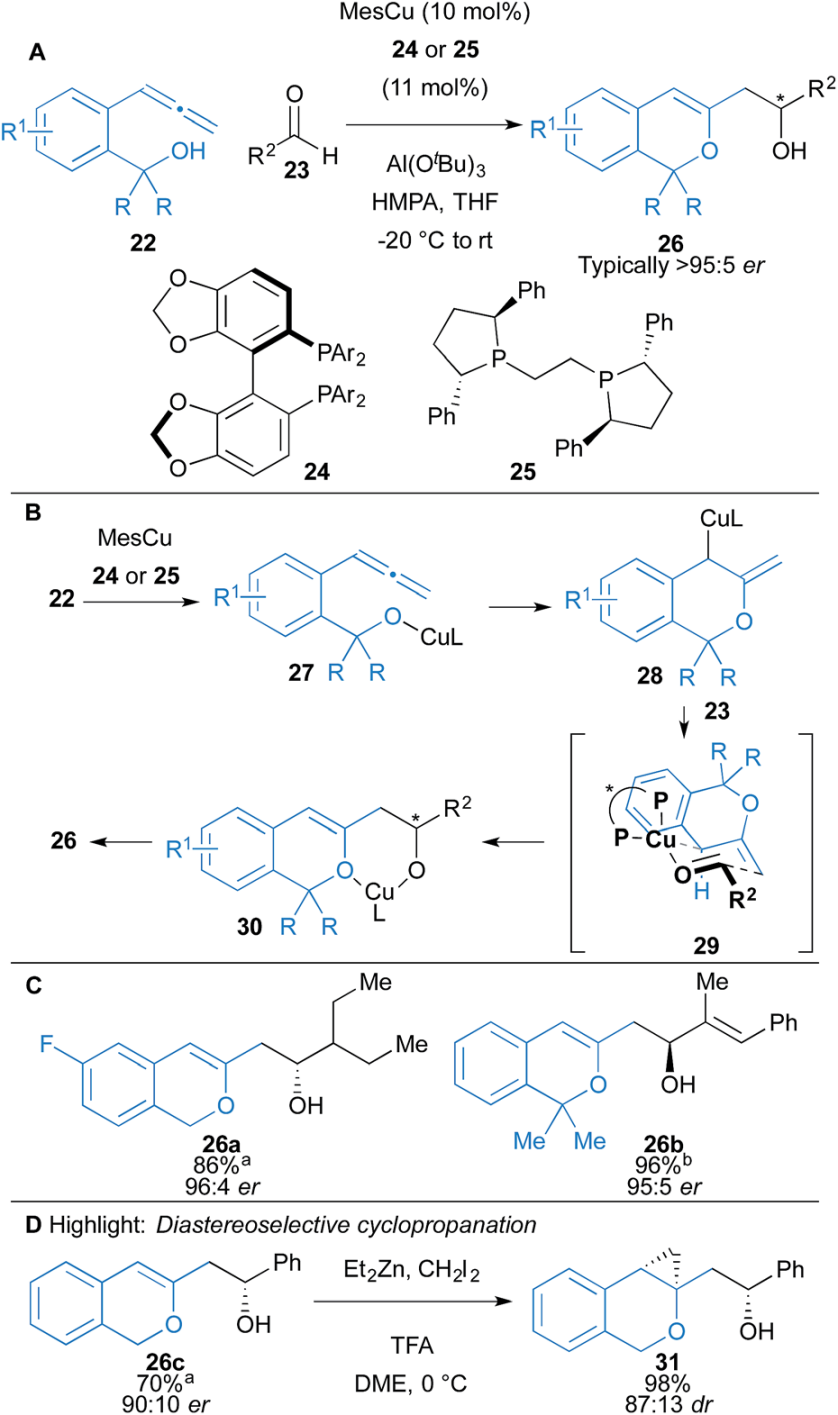
Shimizu and Kanai's intramolecular oxycupration of allenes and subsequent coupling to aldehydes. Ar = 3,5-^*t*^Bu_2_-4-MeO-C_6_H_2_; ^a^ligand **24** was used with Al(O^*t*^Bu)_3_; ^b^ligand **25** was used in the absence of Al(O^*t*^Bu)_3_.

Shimizu, Kanai and co-workers reported an intramolecular oxycupration of allenes **22** (Scheme 4).^18^ The reaction proceeds through an allylcopper intermediate **28**, which then undergoes a subsequent enantioselective addition to aldehydes **23** to afford 1*H*-isochromene derivatives **26** (Scheme 4B). Ligand **24** was used in the case of allenes **22** containing primary alcohols (R = H) and ligand **25** for tertiary alcohols (R = Me). HMPA was found to enhance enantioselectivity and reduce protonation of the allylcopper intermediate, which led to a major side product in some cases. In more challenging substrates, Al(O^*t*^Bu)_3_ was used as a co-catalyst to facilitate liberation of the copper catalyst from intermediate **30**. The reaction was compatible with aromatic and aliphatic aldehydes, and gave secondary alcohols **26** with high enantioselectivity (typically >95 : 5 *er*) (Scheme 4C). Acetophenone was also used, and gave the corresponding tertiary alcohol in 77% yield and 88 : 12 *er*. The 1*H*-isochromene skeleton bears a versatile enol ether moiety that could readily undergo diastereoselective cyclopropanation to form a tricyclic scaffold **31** (Scheme 4D).

**Scheme 5:**
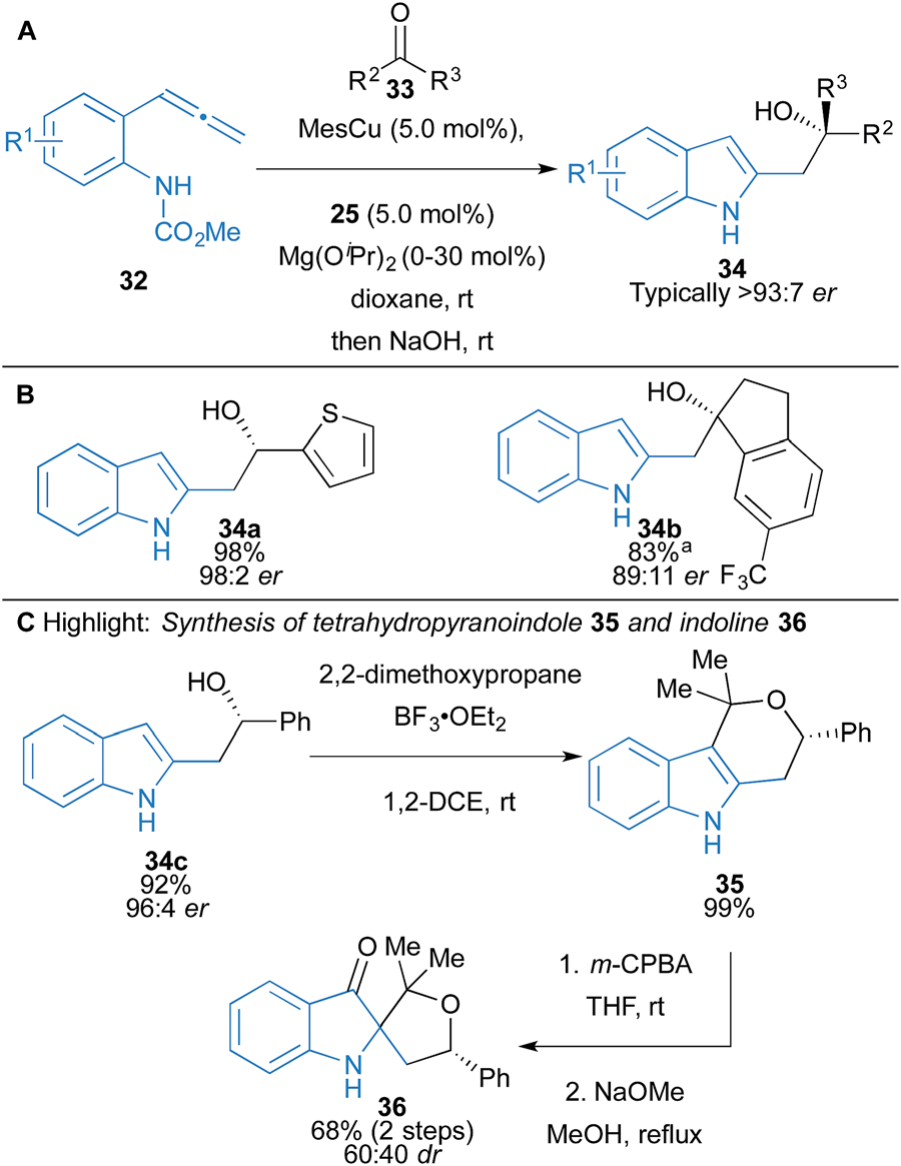
Kanai's intramolecular aminocupration of allenes and subsequent coupling to aldehydes and ketones. ^a^Mg(O^*i*^Pr)_2_ was used.

Kanai and co-workers have further developed an intramolecular amido-cupration of allenes **32** and asymmetric addition to ketones and aldehydes (**33**), to afford substituted indole scaffolds **34** (Scheme 5).^19^ The one-pot process proceeds with high regio-, chemo- and enantioselectivity with a broad substrate scope including addition to aromatic and aliphatic aldehydes and ketones, where ketones afford products bearing tertiary alcohols (Scheme 5B). In some cases, the additive Mg(O^*i*^Pr)_2_ dramatically increased the yield of coupling by decreasing the Brønsted basicity of the allylcopper species, relative to its nucleophilicity, and suppressing the undesired protonation pathway. The synthetic utility of the products was demonstrated using **34c**, which was converted to the pharmacophore tetrahydropyranoindole **35** and indoline **36** (Scheme 5C).

**Scheme 6:**
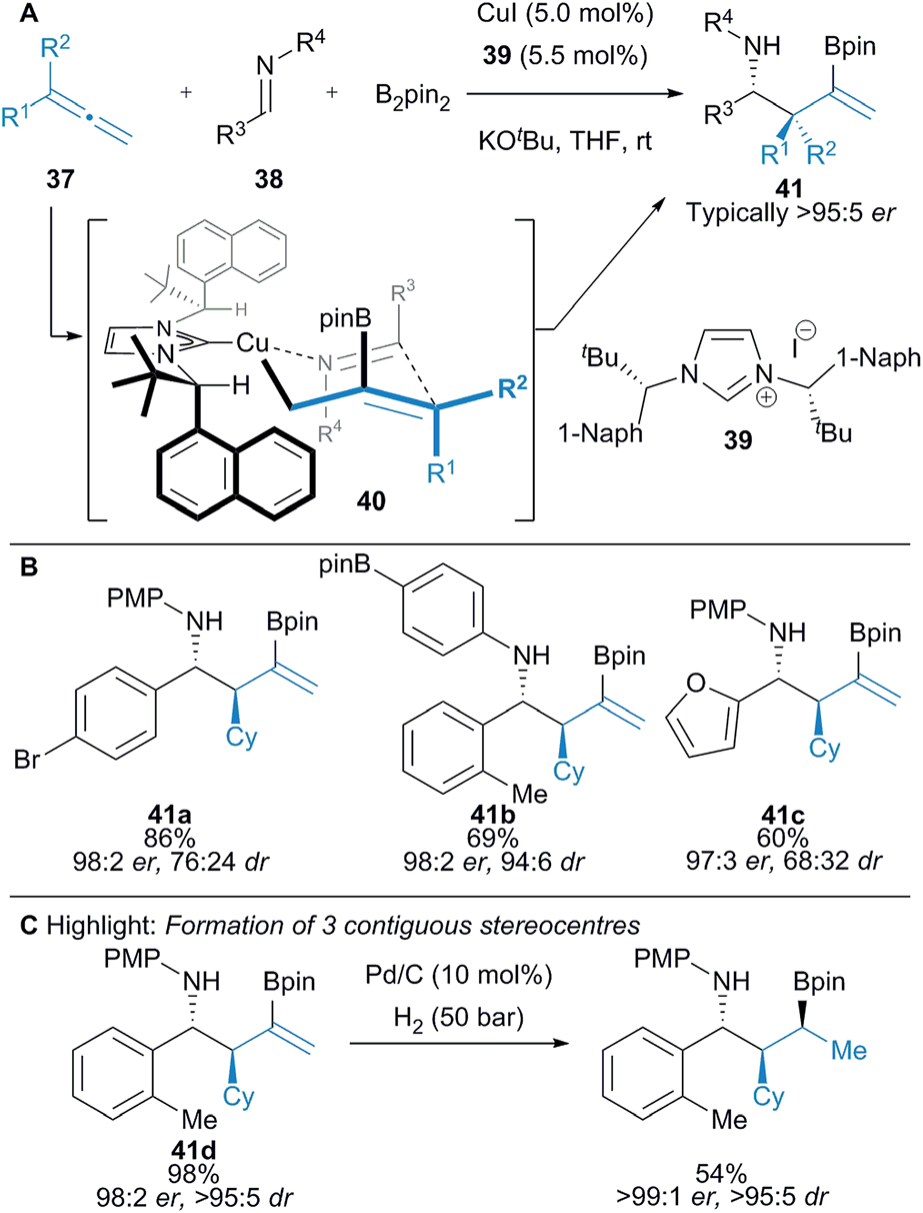
Procter's borocupration of allenes and subsequent coupling to imines. Enantiomeric ratio given for the major diastereomer.

Procter and co-workers reported the enantioselective Cu(i)–NHC catalysed three-component coupling of allenes **37**, B_2_pin_2_ and aldimines **38** that proceeds with high diastereo- and enantiocontrol and excellent functional group tolerance, to yield homoallylic amines **41** bearing adjacent stereocentres (Scheme 6).^20^ Regioselective borocupration of allenes leads to *in situ* formation of an allylcopper intermediate **40**. Computational analysis revealed that the allylcopper addition to imine likely proceeds *via* a chair-like transition state structure **40**, where the substituents on the imine are in pseudoaxial positions (Scheme 6A).^21^ The boronate ester products can be isolated owing to an intramolecular nitrogen–boron interaction. The coupling products **41** could be further functionalised by oxidation to form branched β-amino ketones, and hydrogenated to form secondary alkyl boronates bearing three contiguous stereocentres (Scheme 6C).

**Scheme 7:**
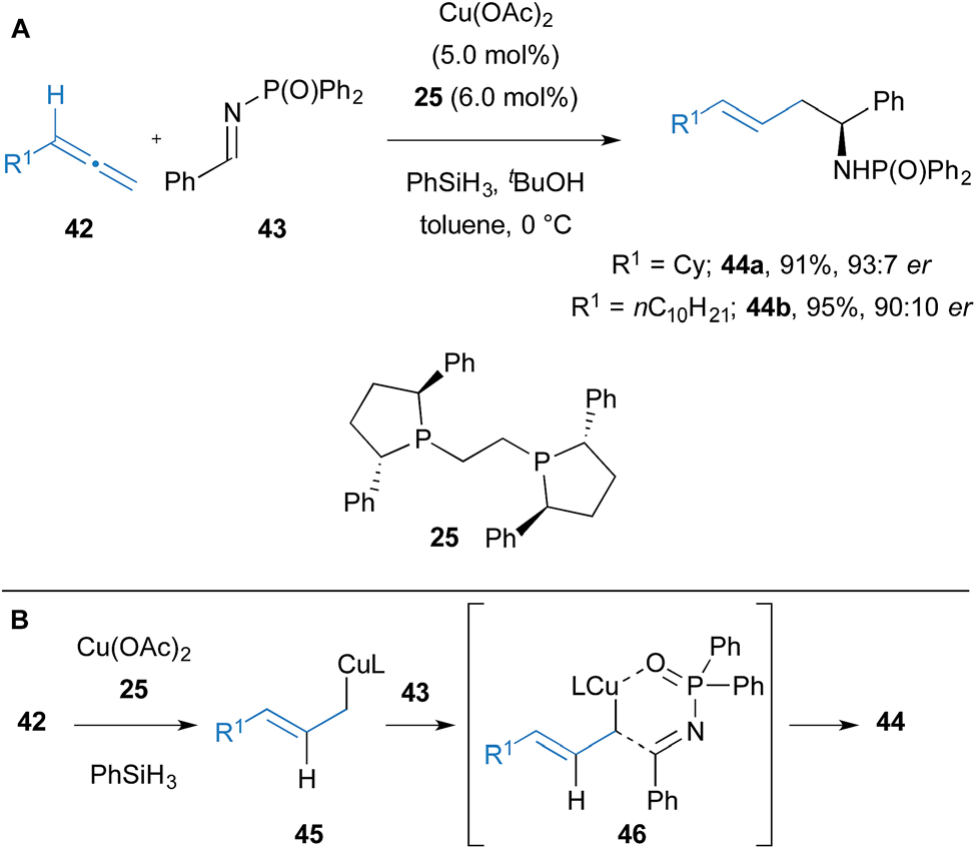
Buchwald's hydrocupration of allenes and subsequent coupling to imines.

Soon after, Buchwald and co-workers reported a Cu(ii)-catalysed regiodivergent and diastereoselective allylation of aldimines **43** to synthesise branched or linear homoallylic amines.^22^ An enantioselective variant of their linear-selective allylation reaction using *N*-diphenylphosphinyl protected imines was demonstrated with two examples (Scheme 7). Hydrosilanes were used to form *in situ* a copper hydride intermediate, which upon hydrocupration of allene **42** and subsequent fast equilibration, afforded the thermodynamically favoured terminal *E*-allylcopper intermediate **45** (Scheme 7B). The allylcopper species reacts with the imine through the α-position, in contrast to the γ-addition of the allylcopper observed in Procter's work. The transition state structure **46** for the allylation of aldimines **43**, supported by DFT studies, was proposed to involve copper coordination to oxygen of the phosphinyl imine, then transfer of the allyl fragment to afford linear products selectively.

**Scheme 8:**
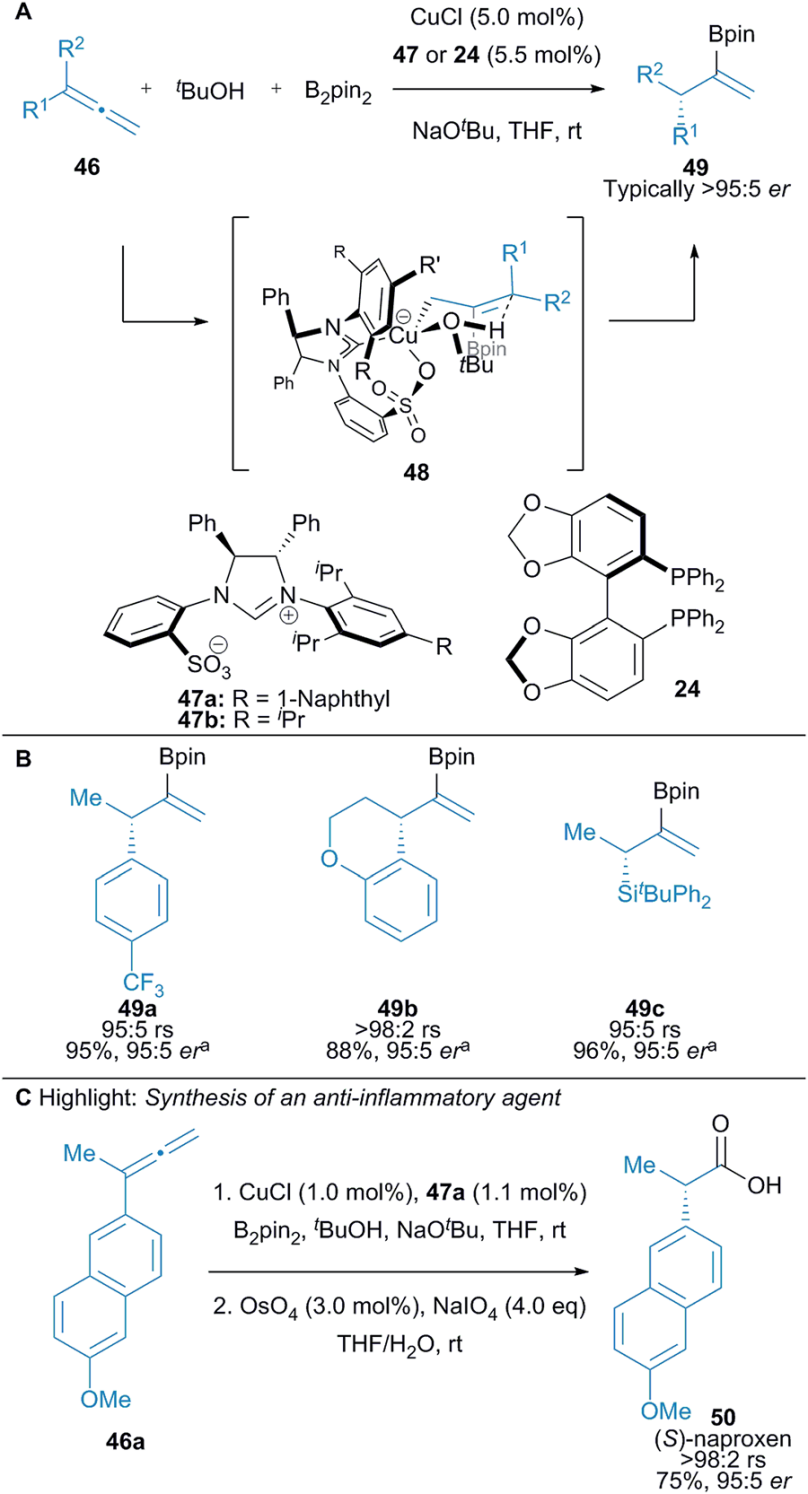
Hoveyda's borocupration of allenes and subsequent protonation of the allyl copper intermediate. rs = regioselectivity (γ/α site selectivity of protonation). ^a^using **47a**.

Hoveyda and co-workers demonstrated the use of prochiral 1,1-disubstituted allenes **46** in a Cu(i)-catalysed protoboration reaction affording vinyl boronates **49**, with high enantioselectivity achieved using either a chiral NHC precursor **47** or chiral phosphine ligand **24** (Scheme 8).^23^ The reaction proceeds *via* γ-protonation of an *in situ* formed allylcopper intermediate (*cf.* **48**) to provide access to enantioenriched vinyl boronates **49** with up to >98% regioselectivity and typically >95 : 5 *er* in excellent yield (Scheme 8B). 1,1-Disubstituted allenes bearing an alkyl and aryl substituent were used. The use of *tert*-butanol as the proton source was crucial for the high enantioselectivity of the reaction. The versatility of vinyl boronates was well-demonstrated with representative procedures for the preparation of enantiomerically enriched vinyl bromides, methyl ketones and carboxylic acids. The methodology was also applied in an enantioselective synthesis of the non-steroidal anti-inflammatory agent, (*S*)-naproxen **50** (Scheme 8C).

**Scheme 9:**
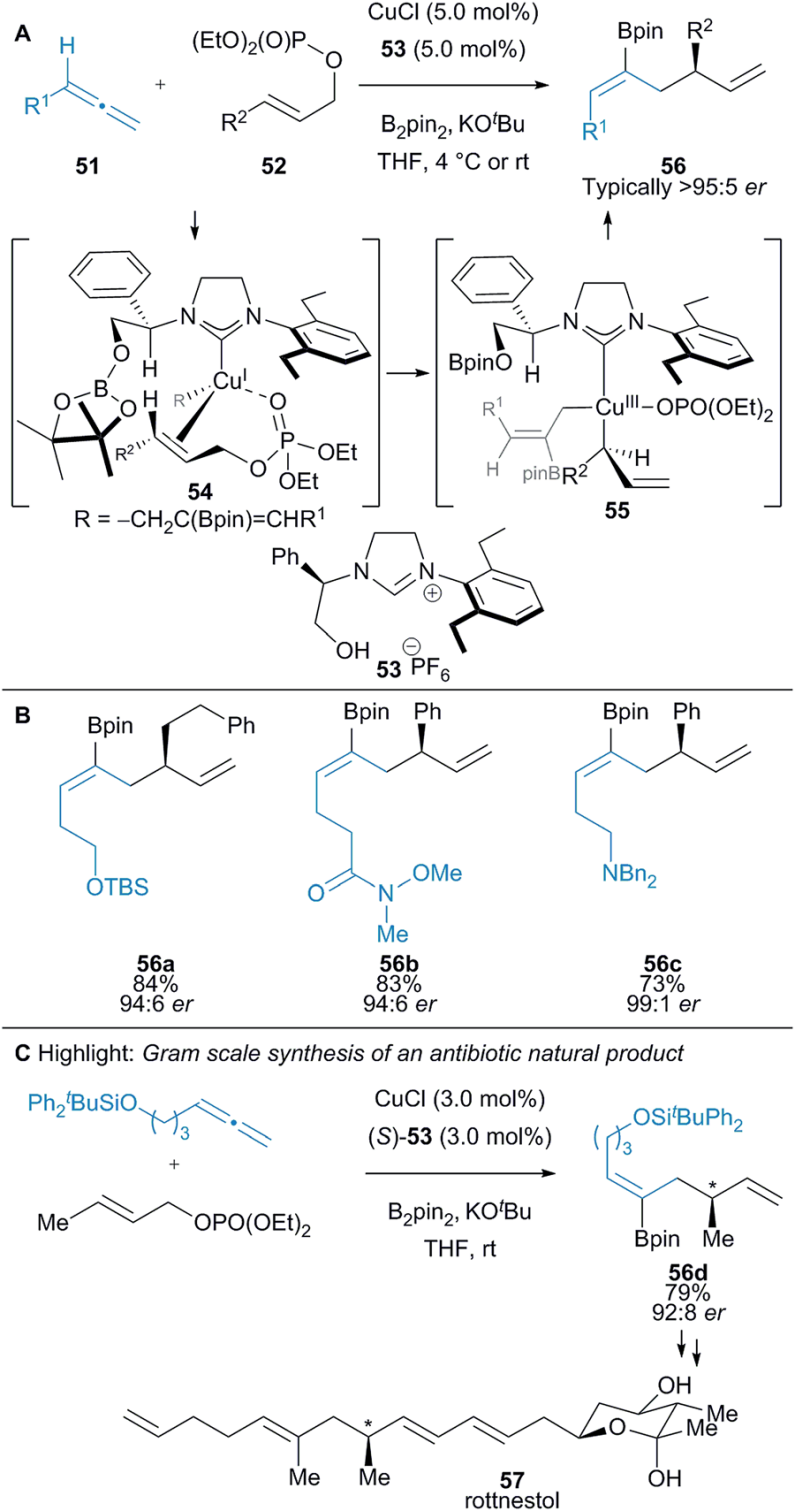
Hoveyda's borocupration of allenes and subsequent coupling with allyl phosphonates.

An enantioselective Cu(i)-catalysed borylative allyl–allyl coupling was successfully developed by Hoveyda and co-workers, using an *in situ* generated allylcopper intermediate (*cf.* **54**) and allylic phosphonates **52** to synthesise 1,5-diene motifs **56** (Scheme 9).^24,25^ Allylic phosphonates can react through a S_N_2′ or S_N_2 pathway and can undergo direct borylation to form a nucleophilic allylic boronate, yet despite this, judicious choice of ligand resulted in excellent chemo-, regio-, and enantioselectivity. The proposed reaction pathway is supported by DFT calculations and involves an allylcopper intermediate that coordinates with the allylic phosphate **52** to form a tetrahedral Cu(i) complex **54**. Oxidative addition in **54** forms square planar Cu(iii) complex **55** which undergoes reductive elimination to afford the desired product **56** (Scheme 9A). Wide functional group tolerance was demonstrated (Scheme 9B) and the methodology applied to the gram-scale synthesis of two natural products, including the antibiotic rottnestol **57** (Scheme 9C).

**Scheme 10:**
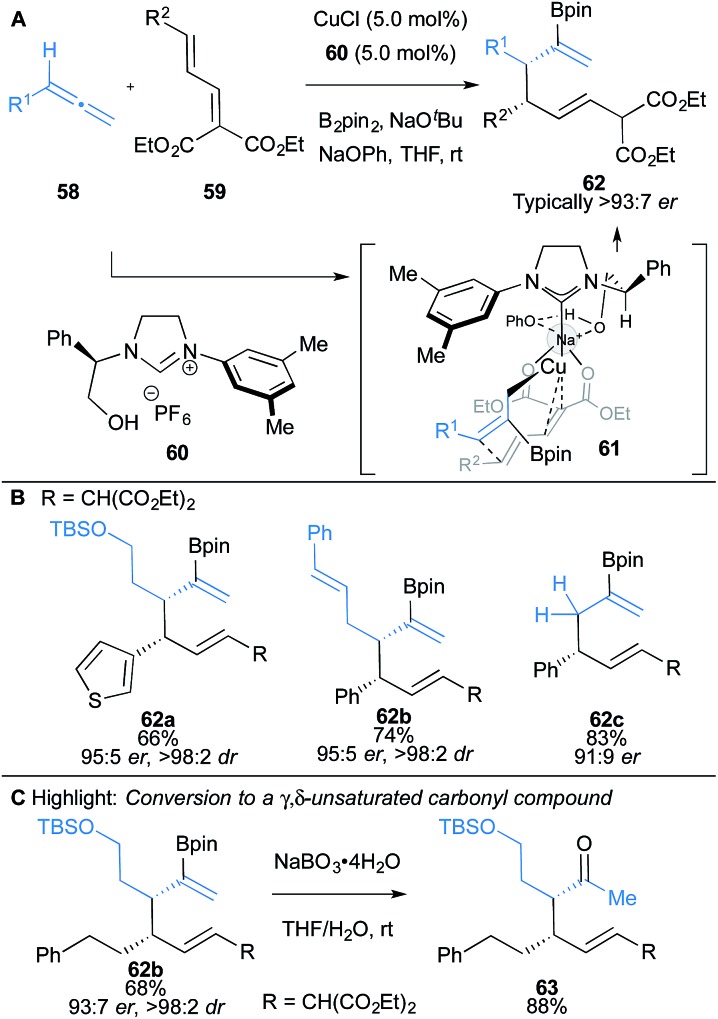
Hoveyda's borocupration of allenes and subsequent coupling to dienoates. Enantiomeric ratio given for the major diastereomer.

Hoveyda and co-workers have also reported a three-component coupling of allenes **58** with dienoates **59** and B_2_pin_2_ leading to highly functionalised 1,5-dienes **62** (Scheme 10).^14*a*^ Excellent regio-, diastereo- and enantioselectivity were achieved using imidazolinium salt **60** bearing an unprotected hydroxyl group, which was found to be essential for enantioselectivity. DFT studies suggested the hydroxy group provides key ionic interactions and hydrogen bonding in the transition state (Scheme 10A). Substrates bearing heterocycles, alkenyl and alkynyl substituents were compatible (Scheme 10B). The enantiomerically-enriched products **62** allow access to γ,δ-unsaturated ketones **63** with vicinal stereocentres, which are otherwise difficult to access directly (Scheme 10C).

## 3 Future prospects in copper catalysed allene functionalisation

In addition to the aforementioned enantioselective processes, there have been reports of the non-enantioselective copper-catalysed functionalisation of allenes that enables access to diversely functionalised molecules that are ripe for the development of enantioselective variants. As well as the silylative variants of the aforementioned borylative couplings of aldehydes,^26^ ketones^27^ and imines,^21^ described above, such copper catalysed functionalisations of allenes include protoboration that selectively delivers alkenyl or allylic boronic esters,^28^ borostannylation that yields β-boryl allyl stannanes,^29^ hydrocupration followed by branch selective imine allylation,^22^ carboboration that produces alkenyl boronic esters,^30^ intramolecular hydroamination for the formation of 3-pyrrolines or 2-alkenylpyrrolidines,^31^ and conjugate addition type processes of allenoates and their derivatives.^32^

**Scheme 11:**
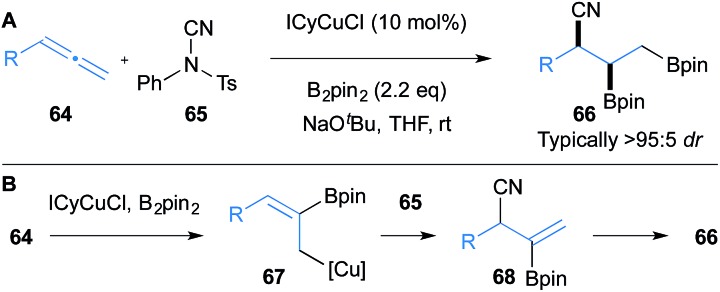
Montgomery's borocupration of allenes and subsequent cyanation. ICyCuCl = chloro[1,3-dicyclohexylimidazol-2-ylidene]copper(i).

In this section we wish to highlight selected recent advances in non-enantioselective copper catalysed processes that deliver densely functionalised molecules derived from allenes. For example, in 2016, Montgomery reported a rare example of a diastereo- and regioselective copper-catalysed trifunctionalisation of terminal allenes **64** (Scheme 11).^33^ In this reaction, initial borocupration of allene **64** leads to allyl copper intermediate **67** that is subsequently cyanated with *N*-cyano-*N*-phenyl-*p*-methylbenzenesulfonamide (**65**) to give intermediate **68**. Further borocupration of **68** and protonation provides the trifunctionalised product **66**. As with many of the processes presented herein, the method displays impressive functional group tolerance.

**Scheme 12:**
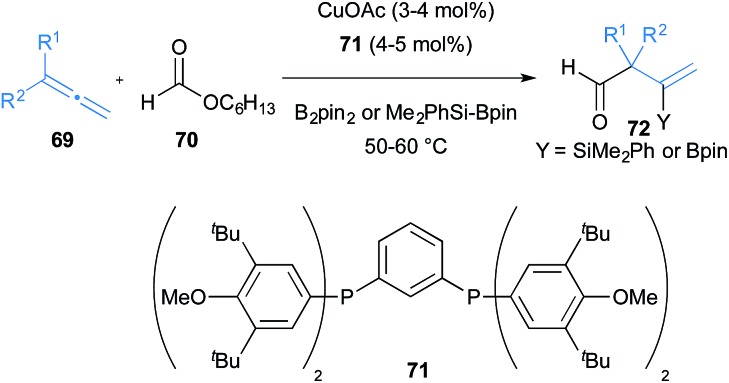
Fujihara and Tsuji's boro- and sila-cupration of allenes and subsequent coupling with formates.

Fujihara, Tsuji and co-workers showed that allenes **69** can be functionalised *via* boraformylation and silaformylation reactions (Scheme 12).^34^ The allyl copper generated after initial boro- or silacupration of 1,1-disubstituted allenes **69** with CuOAc and the bulky diphosphine ligand **71** can be trapped with formate ester **70** to deliver β-boryl or β-silyl β,γ-unsaturated aldehydes **72**. This reaction is particularly impressive as it has the potential to deliver aldehydes containing α-quaternary carbons.

**Scheme 13:**
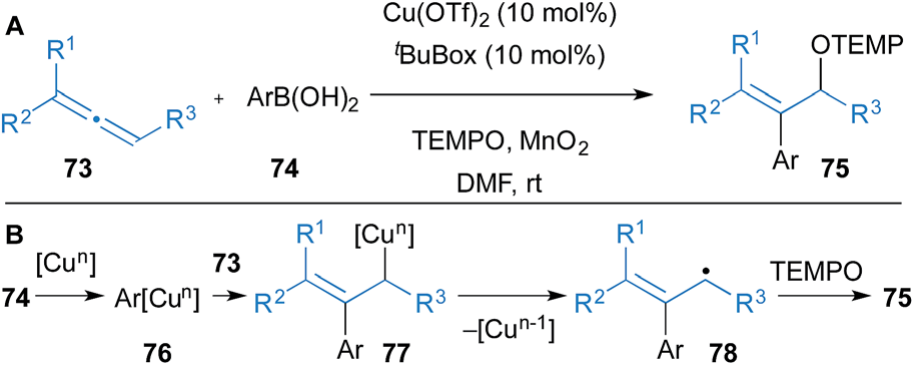
Shimizu and Kanai's carbocupration of allenes and subsequent oxidation. ^*t*^BuBox = 2,2′-isopropylidenebis[(4*S*)-4-*tert*-butyl-2-oxazoline].

Finally, Shimizu and Kanai reported that allenes **73** undergo oxyarylation in the presence of a variety of aryl boronic acids **74** and TEMPO to yield β-arylated allylic alcohol derivatives **75** under copper catalysis (Scheme 13).^35^ Copper(i) salts were also effective in the transformation but Cu(OTf)_2_ was optimal. The mechanism is proposed to go through initial transmetallation of copper with aryl boronic acids to form aryl copper **76** (Scheme 13B). Subsequent carbocupration of allene **73** yields allyl copper **77** that undergoes homolysis to form allyl radical **78** and a reduced copper species. TEMPO traps **78** to form product **75**, and MnO_2_ allows for oxidation of copper to reform the catalyst.

Although these methods are yet to be shaped into enantioselective processes, they yet again show the impressive array of allene functionalisations that are possible under copper catalysis.

## 4 Conclusions

The use of allenes as feedstocks in conjunction with copper catalysis allows for the efficient construction of diversely functionalised, enantioenriched molecules. The general reaction manifold involves initial element cupration of the allene to generate an allyl copper that undergoes a subsequent coupling event. The key allyl copper species usually reacts through the γ-position with the electrophilic coupling partner, although α-addition is also known. In addition, the catalysis generally operates through Cu(i) species, but the formation of Cu(iii), followed by reductive elimination has also been proposed with electrophiles that can oxidatively add to allyl Cu(i) complexes.

The above enantioselective transformations only utilise NHC and bisphosphine ligands as chiral inductors. Given the enormous variety of other chiral ligands that might be employed, such as sulfoxides,^36^ bisoxazolines,^37^ and even chiral counterions,^38^ the door is open for further, more diverse copper catalysed functionalisation of allenes.

We anticipate that further, exciting developments will be discovered with the use of enantiomerically pure allenes,^39^ or chiral racemic allenes, which have not been investigated in this reaction manifold. Enantiomerically pure allenes might be employed in reactions that transfer axial into point chirality with an achiral copper catalyst. In addition, given that the racemisation of allenes with cuprates is precedented,^40^ resolution processes under copper catalysis can also be envisaged.

Currently, enantioselective processes have been developed that involve boro-, sila-, hydro-, carbo-, amino- and oxy-cupration of allenes to generate allyl coppers that are subsequently coupled with aldehydes, ketones, imines, protons, allyls, and unsaturated carbonyls. And yet, we have only scratched the surface. Given the potential variety of initiating element-cuprations, the plethora of coupling partners available, and the possibility of further *in situ* transformations, we can look forward to many more diverse copper catalysed processes that result in the enantioselective mono-, di- and even trifunctionalisation of allenes.
